# Cyclodextrins produced by cyclodextrin glucanotransferase mask beany off-flavors in plant-based meat analogs

**DOI:** 10.1371/journal.pone.0269278

**Published:** 2022-06-03

**Authors:** Kiyota Sakai, Yukihide Sato, Masamichi Okada, Shotaro Yamaguchi

**Affiliations:** Amano Enzyme Inc., Innovation Center, Kakamigahara, Japan; Poznan University of Life Sciences: Uniwersytet Przyrodniczy w Poznaniu, POLAND

## Abstract

The widening gap between the supply and demand for meat products has increased the need to produce plant-based meat analogs as protein sources. Meat analogs are principally composed of soy-based textured vegetable proteins. Despite ongoing technical developments, one of the unresolved challenges for plant-based meat analogs is the off-flavor from soy, which limits their consumer acceptability. Among the various methods developed for overcoming this challenge, masking the beany flavors with cyclodextrins (CDs) is an attractive, cost-effective, and safe strategy. However, the current established CD treatment method does not meet the requirement for a clean-label. This study aimed to develop more acceptable off-flavor-masking technologies for plant-based patties for modern clean-label preferences using enzymatic methods. We used the cyclodextrin glucanotransferase (CGT), “Amano,” as a commercially available food-grade CGT. The CGT-catalyzed reaction in plant-based patties yielded 17.1 g/L CD. As CGT could yield sufficient CD in the patties, we investigated whether CDs produced by CGT could mask the off-flavors released from the plant-based patties. The CGT-treated patties had significantly lower volatilization amounts of the known beany off-flavor-generating compounds compared to the non-treated patties. Moreover, CGT treatment improved the texture of the patties and increased their water- and oil-holding capacity. As CGT is rendered inactive after cooking, it would not be considered an additive. These findings indicated that CDs produced by the CGT reaction could effectively mask off-flavors of meat analogs and improve their physical properties while meeting clean-label requirements.

## Introduction

The widening gap between the supply and demand for meat has increased the need to produce plant-based meat analogs as protein sources [1]. It has been estimated that the progression from current omnivorous diets to vegan and ovo-lacto vegetarian diets can decrease greenhouse gas emissions by approximately 50% and 35%, respectively [2]. In addition, numerous studies have reported health benefits associated with replacing animal sources of protein with plant-based proteins, including reduced risks of type 2 diabetes, heart disease, and stroke [3, 4]. Therefore, developing better plant-based diets would address the current protein crisis and positively impact the environment and human health [1].

Meat analogs are principally composed of textured vegetable proteins (TVPs) that imitate the fibrillar structure of meat muscle [5]. Soybean proteins are widely used for the production of TVPs. Soy-based TVP is a plant-based protein product that is cholesterol-free, with low concentrations of saturated fat and high concentrations of essential amino acids [6, 7]. Despite ongoing technical developments, the appearance, flavor, taste, and texture of plant-based meat analogs differ from those of traditional meat products [1, 8, 9]. One of the unresolved challenges for plant-based meat analogs is their flavor, particularly the off-flavors from soy [1, 8, 9]. As soybean protein has an unpleasant beany and grassy odor, it affects the overall flavor of meat analogs, limiting consumer acceptability and restricting the development of meat analogs as food products [10].

The beany flavor is a combination of more than 20 volatile compounds produced during soybean growth and processing of soybean [11, 12]. These compounds can be mainly divided into fatty aldehydes, fatty alcohols, fatty ketones, furans, furan derivatives, and aromatic compounds [13]. Among these, hexanal, 1-octen-3-ol, and benzaldehyde are considered the compounds that primarily contribute to the off-flavor [12]. Currently, there are three main approaches for the removal of soybean beany flavor: genetic engineering, physical, and chemical methods [12]. However, these approaches cannot solve the technical challenges associated with beany off-flavors.

Cyclodextrins (CDs) are cyclic oligosaccharides composed of six or more α-1,4-linked glucose units that are also industrially produced from starch using the enzyme cyclodextrin glucanotransferase (CGT, EC 2.4.1.19). CDs with 6, 7, and 8 α-glucose residues are called α-CD, β-CD, and γ-CD, respectively. CDs can form inclusion complexes with hydrophobic chemicals as they possess a hydrophilic exterior and a hydrophobic interior [14]. Therefore, CDs are used to remove cholesterol from dairy and egg products, decaffeinate coffee, and for flavor encapsulation [14]. Moreover, CD-mediated masking or reduction of undesirable flavors has shown considerable promise in food applications [12, 14]. In a previous study, the addition of CDs to soymilk decreased the concentration of volatile compounds associated with beany flavors [15]. Zhu and Damodaran (2018) reported the volatile compound removal properties of CDs on soy protein isolates [16]. However, masking the beany flavors of soy-based TVP and meat analogs using CDs has not been extensively studied.

CDs were assigned a “generally recognized as safe” (GRAS) status by the U.S. Food and Drug Administration (US-FDA) and an E number by the European Food Safety Authority (EFSA) and are considered as safe food additives [17, 18]. Therefore, CD-mediated masking or reduction of beany flavors is an effective and safe approach. However, the increasing consumer demand for clean-label food demands the reduction of additives, and the scientific community and food industry are developing more acceptable strategies for decreasing the beany flavor of plant-based meat analogs [19]. Among these, using food-grade enzyme catalysis is an attractive tool as enzymes do not need to be listed as an additive, provided that they are inactive in the finished product (e.g., denatured, inactivated enzymes). Therefore, the masking effect of CGT-produced CDs could be an attractive tool for treating clean-label foods. However, the masking efficiency of CDs produced by the CGT reaction within foods has not been extensively studied.

This study investigated the masking effects of CDs produced using CGT on meat analogs composed of soy-based TVP. CGT treatment effectively reduced the volatilization amounts of the known beany off-flavor-producing compounds compared to the non-treated patties. Moreover, CGT treatment improved the texture of the patties and increased their water- and oil-holding capacity. These findings indicated that CDs produced by the CGT reaction could effectively mask off-flavors of meat analogs and improve their physical properties while meeting clean-label requirements.

## Materials and methods

### Materials

Granule-type soy-based TVP was purchased from Marukome Co. Ltd. (Nagano, Japan). CGT (Amano Enzyme Inc., Nagoya, Japan) is a commercially available food-grade product. α-CD, β-CD, and γ-CD were obtained from FUJIFILM Wako Pure Chemical Corporation (Osaka, Japan). Potato starch was purchased from Nippon Star Chemical Co. Ltd. (Osaka, Japan). Hexanal, heptanal, octanal, nonanal, 2-octenal, 2-nonenal, benzaldehyde, 1-hexanol, 1-octen-3-ol, pyrazine, acetic acid, 2-pentylfuran, and furfural were obtained from FUJIFILM Wako Pure Chemical Corporation (Osaka, Japan).

### Enzyme assays

The activity of CGT was assayed in 1.0-mL reaction mixtures containing 100 mM sodium phosphate (pH 6.0) and 1.0% (w/v) potato starch at 20–80°C for 2 h. The reaction was stopped by incubating at 100°C for 5 min. After stopping the reaction, the reaction mixture was mixed with 0.005% iodine in 0.05% potassium iodide, and the absorbance was measured at 660 nm.

### Preparation for plant-based meat analogs

The base of the TVP matrix was prepared using TVPs and a binder (methylcellulose (MC)), followed by the addition of water, olive oil, potato starch, and CGT (S1 Table). First, 10 g of dried TVP was immersed in 15 mL water for 30 min for hydration. After hydration, the swollen TVP was mixed with 2.0% MC to obtain the final concentration. Thereafter, 10 g water, 3 g olive oil, 2.5 g soy protein isolates, and 1.0 g potato starch were added to 25 g TVP matrix. The samples were blended for 60 s using a hand blender. Subsequently, 100 U/g-starch CGT was added to the TVP matrix and blended for 60 s. The CGT used in this study was a commercially available food-grade product. The TVP matrix was molded (60 mm × 40 mm area × 25 mm height) and incubated at 60°C for 2 h. The matrix was then cooked at 150°C for 15 min and cooled to room temperature (20–25°C) before being used for further analysis.

### High-performance liquid chromatography (HPLC) analysis for CDs

The CDs in the reaction solution and patty matrix were analyzed by HPLC (Shimadzu, Kyoto, Japan). To extract CDs, the patty matrix was crushed using a mixer. The solutions containing various maltooligosaccharides and dextrin were added to 80% methanol and centrifugated at 15,000 *× g* for 10 min to precipitate saccharides with a molecular weight greater than oligosaccharides. Then, these solutions were incubated with 2.0 mg/mL glucoamylase (Sigma-Aldrich, St. Louis, USA) at 60°C for 3 h to hydrolyze all oligosaccharides, except CDs. After filtering through a membrane filter (pore size 0.45 μm), the supernatant containing CDs was injected into the HPLC. The supernatant was separated on an MCI GEL CK04S (300 × 8.0 mm I.D.; Mitsubishi Chemical Corporation, Tokyo, Japan) connected to an evaporative light scattering detector system, with MilliQ water for 20 min at a flow rate of 0.4 mL/min at 80°C. Standard curves were prepared using solutions containing different concentrations of CD.

### Headspace solid-phase microextraction-gas chromatography/mass spectrometry (HS-SPME-GC/MS)

Finely cut mold matrix (3 g) was added to 20 mL screw glass vials, and HS-SPME-GC/MS was performed using an autosampler (Shimadzu, Kyoto, Japan). 1,2-Dichlorobenzene (1.0 μL/g-sample) was added as an internal standard to check the retention time of different samples. Polydimethylsiloxane (PDMS; Shimadzu, Kyoto, Japan) and divinylbenzene (DVB)/PDMS (Shimadzu, Kyoto, Japan) were used as SPME fibers for extraction. The fiber was conditioned at 250°C for 30 min before each use. The vials were incubated for 10 min at 80°C in an autosampler with agitation (270 rpm). Volatile compounds were extracted by placing each fiber in a vial and exposing it to the headspace for 30 min at 80°C with agitation (270 rpm). The fiber was removed and inserted into the GC injection port. Desorption time for the SPME fiber injection was 3 min at 250°C. A gas chromatography system (GC-2030, Shimadzu, Kyoto, Japan) attached to a triple quadrupole MS (Shimadzu) was used. The injection port used was in split mode with a flow rate of 60 mL/min and a surge pressure of 100 kPa. Helium was used as the carrier gas at a constant flow rate of 1 mL/min. An InterCap Pure-WAX column (30 m × 0.25 mm, film thickness 0.25 μm, GL Sciences Inc., Tokyo, Japan) was used for volatile compound separation. The oven temperature was programmed according to the manufacturer’s application datasheet for this column, with slight modifications. The initial oven temperature was maintained at 40°C for 0.5 min. The temperature was then raised at 2°C/min to 120°C and finally at 10°C/min to 230°C, where it was kept constant for 5 min. The ion source temperature was 200°C, and the MS range was set to 29 to 450 m/z. The volatile compounds were identified by comparing the results with the mass spectra and retention indices from the spiked internal standard and a database developed using the National Institute of Standards and Technology library guidelines. To quantify volatile compounds, standard curves were generated from plant-based patties spiked to contain 0.1-, 1.0-,5.0-, and 10-μg standard/g-sample for each volatile compound. Under the same conditions, these spiked samples were analyzed by HS-SPME-GC/MS.

### Sensory evaluation

The smell and taste of plant-based patties were evaluated by five employees of Amano Enzyme Inc. trained in sensory evaluation. For taste tests, the samples were evaluated for five attributes (sweetness, sourness, saltiness, bitterness, and umami). For smell tests, the samples were evaluated for two attributes (odor and fragrance). The samples were scored on a scale from 1 (weak) to 5 (strong).

### Measurements for texture profile analysis (TPA) of patties

TPA was performed using COMPAC-100II (Sun Scientific Co., Ltd., Tokyo, Japan) equipped with a cylindrical probe with an area of 31.4 mm [20]. After grilling, the meat analogs were prepared for analysis and cut to a length of 15 mm to obtain homogeneous extrudates. The diameter of each extrudate is approximately 20 mm. A double-compression cycle was performed at 1 mm/s until a recorded deformation of 50% was achieved. The following parameters were evaluated: hardness, maximum force recorded during the first compression; cohesiveness, area of work during the second compression divided by the area of work during the first compression; springiness, the distance recorded during the second compression divided by the distance of the first compression; and chewiness, hardness × cohesiveness × springiness.

### Cooking loss

The cooking method and conditions were determined based on a previous study [21]. The patties were cooked at 150°C for 15 min, or until the temperature at the center of the patty reached 80°C. After cooking, samples were cooled to room temperature (20–25°C). Cooking loss was calculated as the percentage weight difference between the patty before cooking and after cooking, using the following formula: cooking loss (%) = [(W_1_ − W_2_)/W_1_] × 100, where W_1_ is the weight of the patty before grilling (g), and W_2_ is the weight of the patty after grilling (g).

## Results and discussion

### Biochemical characterization of food-grade CGT

**Fig 1:**
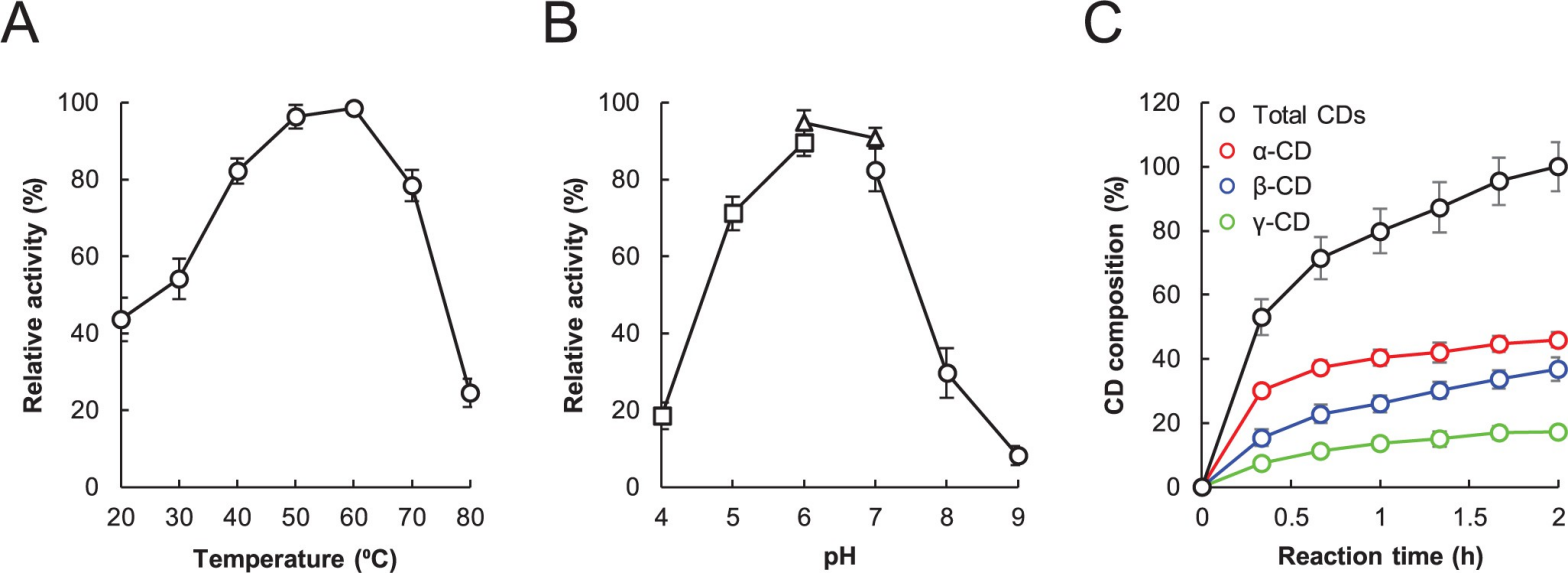
Biochemical characterization of food-grade cyclodextrin glucanotransferase (CGT). (A) Optimal temperature for CGT activity. Enzyme reactions proceeded at temperatures ranging from 20–80°C. (B) Optimal pH for CGT activity. Enzyme reactions proceeded over a pH range of 4.0–9.0 in 50 mM sodium acetate (pH 4.0–6.0, square), 50 mM sodium phosphate (pH 6.0–7.0, triangle), and 50 mM Tris–HCl (pH 7.0–9.0, circle). (C) Composition of cyclodextrin produced from potato starch through CGT reaction. Data are presented as the mean ± standard deviation of three experiments.

In this study, we used the cyclodextrin glucanotransferase “Amano” as the commercially available food-grade CGT. We first investigated the optimal temperature and pH of this CGT for the production of CD from starch. The optimal temperature and pH for CGT production were determined using potato starch. The optimal temperature of CGT was 60°C, with a preferred temperature range (>80%) between 40°C and 60°C (Fig 1A). The optimal pH was 6.0, with a preferred pH range (>80%) of 6.0 and 7.0 (Fig 1B). Next, under optimum conditions (60°C, pH 6.5), the CGT-catalyzed conversion rate of each CD was measured by HPLC (data in S1 Fig). The total CD conversion yield by the 2 h CGT-catalyzed reaction was 17.4 g/L (Fig 1C). The amounts of α-CD, β-CD, and γ-CD produced increased in a time-dependent manner, reaching 4.0 g/h (46.0%), 3.2 g/h (36.8%), and 1.5 g/h (17.2%), respectively.

The production ratios of α-CD, β-CD, and γ-CD were strongly affected by the origin of the CGT. The food-grade CGT used in this study is secreted by *Paenibacillus macerans*. Previous reviews have summarized the production ratio of CDs by CGTs obtained from various microorganisms: 96.5% α-CD obtained using CGT from *Klebsiella oxytoca*, 95% α-CD obtained using CGT from *Thermoanaerobacter*, 51% α-CD, and 32% β-CD obtained using CGT from *Clostridium* spp., 30% α-CD and 51% β-CD obtained using CGT from *Bacillus circulans*, and 36% β-CD, and 61% γ-CD obtained using CGT from *Brevibacillus spp* [22, 23]. Compared to these studies, the CGT used in this study produced α-CD and β-CD more evenly (Fig 1).

### Effects of CDs produced by CGT on beany off-flavors of the meat analog patties

Next, we investigated whether CGT could produce sufficient CDs in plant-based meat analog patties. All the ingredients used in this study were of food-grade. After treating the patties containing starch with CGT, CDs generated by the enzymatic reaction of CGT were extracted from the patties and quantified. The plant-based patty pH level was 6.48 ± 0.02. As a result, the production of α-CD, β-CD, and γ-CD reached 8.5, 5.8, and 2.8 g/L, respectively. The total yield of CD was 17.1 g/L. These findings showed that CGT could produce sufficient CD yield in the plant-based meat analog patty matrixes.

**Fig 2:**
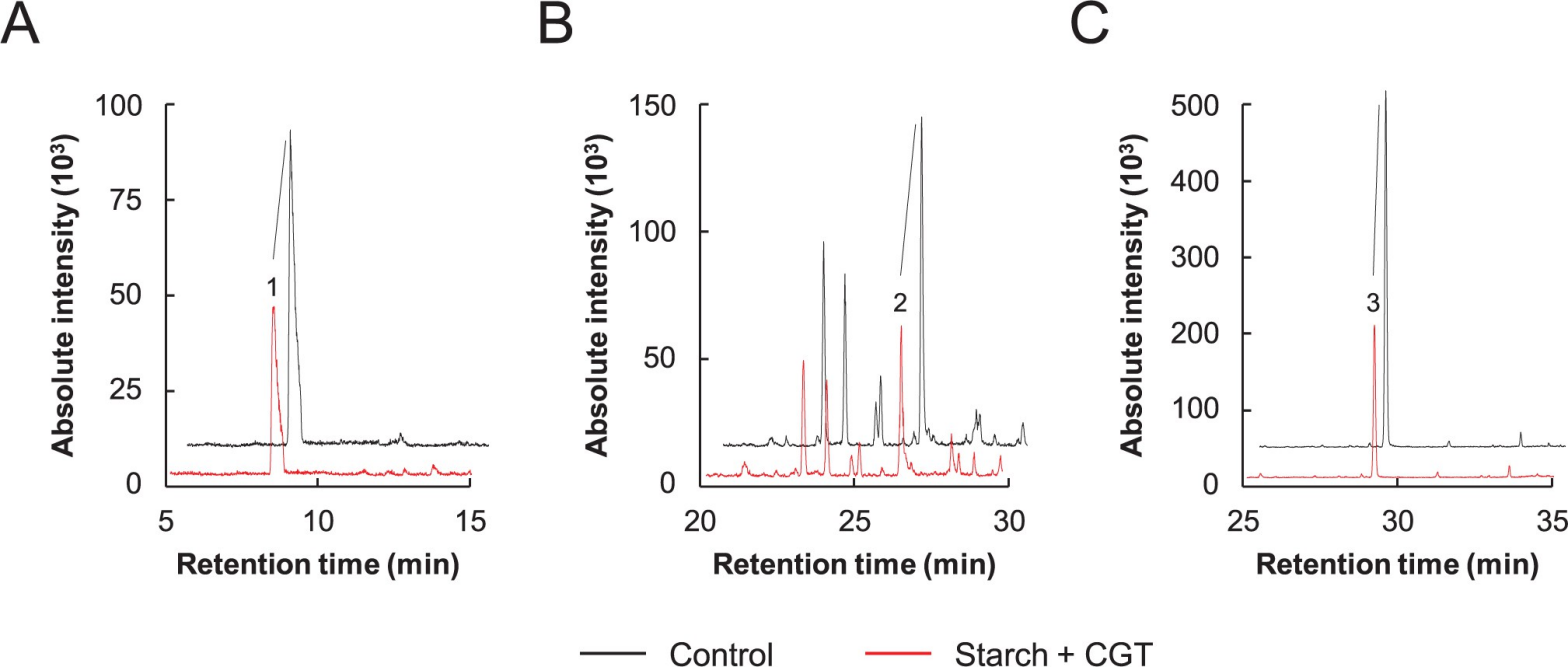
Detection of beany off-flavors in the meat analog patties using headspace-gas chromatography/mass spectrometry (HS-HPME-GC/MS). n-Hexanal (A), 1-octen-3-ol (B), and benzaldehyde (C) were detected by HS-GC/MS in the SIM mode. The major fragment ions of these compounds were 56 (A), 57 (B), and 77 (C) m/z.

Next, we investigated whether the CDs produced by CGT could mask the off-flavors released from the plant-based meat analog patties. After molding and grilling, the volatile components released from each meat analog patty were measured using HS-HPME-GC/MS in the SIM mode. Fig 2 shows the chromatographic analysis of n-hexanal, 1-octen-3-ol, and benzaldehyde, the major off-flavor-producing compounds. The major fragment ions of these compounds were at 56 (Fig 2A), 57 (Fig 2B), and 77 m/z (Fig 2C). Interestingly, volatilization amounts of the three compounds released from CGT-treated patties containing starch were lower than those released from non-treated patties (Fig 2). Other volatile components, generally known as beany off-flavors, were analyzed according to Wang et al. (2021) [12]. Table 1 shows the volatilization amounts of 12 beany off-flavor-producing compounds released from each plant-based meat analog patty. Volatilization amounts of hexanal, heptanal, nonanal, benzaldehyde, octanal, hexanol, 1-octen-3-ol, 2-pentylfuran, and furfural released from CGT-treated patties containing starch were lower compared to that released from the other three patties. These findings indicate that CDs produced by CGT can mask the beany off-flavors released from plant-based meat analog patties.

**Table 1:** Volatile components released from meat analog patties with and without CGT and starch. Data are presented as the mean ± standard deviation of three experiments.

Volatile components	RT (min)	Starch	CGT	Starch + CGT
*Aldehyde*				
Hexanal	8.43	1.09 ± 0.09^a^	1.12 ± 0.07^a^	0.52 ± 0.14^b^
Heptanal	12.11	0.98 ± 0.04^a^	1.06 ± 0.09^a^	0.58 ± 0.18^b^
Octanal	31.11	1.17 ± 0.09^a^	0.93 ± 0.08^a^	0.58 ± 0.13^b^
Nonanal	17.15	1.12 ± 0.03^a^	1.07 ± 0.11^a^	0.63 ± 0.09^b^
2-Octenal	17.37	1.13 ± 0.12^a^	1.06 ± 0.09^a^	1.02 ± 0.06^a^
2-Nonenal	29.89	1.06 ± 0.07^a^	0.94 ± 0.04^a^	1.12 ± 0.22^a^
Benzaldehyde	29.17	1.12 ± 0.05^a^	1.21 ± 0.14^a^	0.67 ± 0.10^b^
*Alcohol*				
1-Hexanol	21.47	0.98 ± 0.10^a^	1.14 ± 0.15^a^	0.61 ± 0.21^b^
1-Octen-3-ol	26.52	1.07 ± 0.09^a^	1.15 ± 0.13^a^	0.55 ± 0.07^b^
*Heterocyclic compound*				
Pyrazine	19.16	1.04 ± 0.03^a^	0.97 ± 0.14^a^	1.12 ± 0.08^a^
*Acid*				
Acetic acid	26.23	0.98 ± 0.21^a^	1.15 ± 0.16^a^	0.95 ± 0.24^a^
*Furan*				
2-Pentylfuran	13.96	1.15 ± 0.05^a^	1.11 ± 1.06^a^	0.48 ± 0.19^b^
Furfural	26.74	1.21 ± 0.24^a^	1.09 ± 0.14^a^	0.63 ± 0.12^b^

Relative values of volatile components from each analog patty compared with those from the control patty are shown. Data are presented as mean values ± standard deviation of three independent experiments. Different letters in the same row mean significant differences at

*p<0.05. CGT, cyclodextrin glucanotransferase; RT, retention time of each compound.

The binding affinity of CDs for nonpolar compounds is related to their cavity size [24]. It has been reported that α-CD forms complexes with aliphatic chains, β-CD forms complexes with aromatic and heterocyclic compounds, and γ-CD binds larger molecules, such as steroids [24, 25]. The CGT used in this study mainly produced α-CD and β-CD (Fig 1C), and the volatilization amounts of various fatty alcohols, fatty aldehydes, and aromatic compounds decreased (Table 1). The ability of the different CDs to mask beany flavors is not well-studied. Therefore, we investigated the effect of α-CD, β-CD, and γ-CD on the efficiency of masking beany off-flavors. As shown in Table 1, CGT produced 1.7% CDs in soy-based patties. Based on this, each CD was added to the soy-based patties at a final concentration of 2%, and all patties were grilled and analyzed under the same conditions (Table 2). The ability to mask fatty aldehydes (hexanal, heptanal, octanal, and nonanal) and fatty alcohols (1-hexanol and 1-octen-3-ol) was in the order α-CD > β-CD > γ-CD. The ability to mask benzaldehyde, 2-pentylfuran, and furfural decreased in the order β-CD > α-CD > γ-CD. Interestingly, none of the CDs decreased the volatilization amounts of unsaturated aldehydes such as 2-octenal and 2-nonenal. These findings indicated that the binding affinity to off-flavors was different among α-CD, β-CD, and γ-CD, and that α-CD and β-CD could effectively mask off-flavors volatilized from soy-based patties.

**Table 2:** Volatile components released from meat analog patties with and without each CD. Data are presented as the mean ± standard deviation of three experiments.

Volatile components	RT (min)	α-CD	β-CD	γ-CD
*Aldehyde*				
Hexanal	8.43	0.48 ± 0.09^c^	0.78 ± 0.12^b^	1.12 ± 0.05^a^
Heptanal	12.11	0.55 ± 0.21^c^	0.79 ± 0.15^b^	1.09 ± 0.05^a^
Octanal	31.11	0.61 ± 0.06^b^	0.84 ± 0.14^a^	1.05 ± 0.06^a^
Nonanal	17.15	0.59 ± 0.05^b^	0.83 ± 0.05^a^	0.98 ± 0.12^a^
2-Octenal	17.37	1.09 ± 0.07^a^	0.96 ± 0.11^a^	1.12 ± 0.20^a^
2-Nonenal	29.89	1.16 ± 0.12^a^	1.13 ± 0.07^a^	1.10 ± 0.11^a^
Benzaldehyde	29.17	0.84 ± 0.12^ab^	0.63 ± 0.08^b^	1.17 ± 0.13^a^
*Alcohol*				
1-Hexanol	21.47	0.54 ± 0.13^b^	0.86 ± 0.06^a^	0.98 ± 0.22^a^
1-Octen-3-ol	26.52	0.62 ± 0.09^b^	0.83 ± 0.12^a^	1.07 ± 0.11^a^
*Heterocyclic compound*				
Pyrazine	19.16	1.07 ± 0.12^a^	1.10 ± 0.02^a^	0.97 ± 0.05^a^
*Acid*				
Acetic acid	26.23	1.06 ± 0.25^a^	0.94 ± 0.20^a^	0.98 ± 0.14^a^
*Furan*				
2-Pentylfuran	13.96	0.72 ± 0.16^ab^	0.53 ± 0.22^b^	1.07 ± 0.17^a^
Furfural	26.74	0.90 ± 0.05^a^	0.67 ± 0.04^b^	1.02 ± 0.07^a^

Relative values of volatile components from each meat analog patty compared with those from the control patty are shown. Data are presented as mean values ± standard deviation of three independent experiments. Different letters in the same row mean significant differences at

*p<0.05. CD, cyclodextrin; RT, retention time of each compound.

In this study, α-CD effectively removed fatty alcohols/aldehydes, and β-CD removed benzaldehyde (Table 2). This difference in the affinity of CDs for nonpolar compounds is related to the size of the cavity. The hydrophobic inner cavity of the α-CD interacts with the acyl chain of the alcohol/aldehyde, resulting in the tight-fitting of an acyl chain with a 4.0 Å diameter (cross-section) within the 4.7–5.3 Å cavity of the α-CD. In contrast, the β-CD cavity interacts with the aromatic ring of benzaldehyde, leading to the tight-fitting of the ring (5.8–6.3 Å) within the 6.0–6.5 Å cavity of β-CD [26]. These interactions could enhance van der Waals forces, further enhancing the interaction energy of the complex [16]. In contrast, the CDs could not eliminate unsaturated aldehydes with shorter hydrophobic chains (Table 1). This is because the binding affinity of CDs to acyl chains is generally affected by the chain length of the fatty alcohol/aldehyde [27]. Moreover, γ-CD was unable to interact with alcohol/aldehyde, benzaldehyde, and other volatile compounds in soy-based patties (Table 2). This is because its cavity size (7.5–8.3 Å) is larger than these volatile compounds, resulting in poor van der Waals and hydrophobic interaction [28]. These results indicated that the presence of α-CD and β-CD, but not γ-CD, was sufficient to mask any off-flavors. Therefore, the CGT used in this study was suggested to be suitable for masking beany off-flavors from soy-based foods, as it mainly produces α-CD and β-CD.

Beany flavors are volatilized not only from soy but also from pea, faba, mung, chickpea, and lentil bean [29–33]. Recently, bean proteins have been developed as plant-based meat analogs. However, various off-flavor-producing compounds, including hexanal, heptanal, 2-octenal, 1-octen-3-ol, and benzaldehyde, were detected and linked to an unpleasant beany and grassy odor in the proteins isolated from these beans. It is known that most bean proteins used for producing plant-based meat analogs release the same off-flavors [30, 34]. These compounds affect the overall flavor of the final product [32, 34]. This phenomenon limits the consumer acceptability of the products [1, 8, 9]. CDs are directly added to bean-based foods to mask the off-flavors [15, 16]. In this study, the CDs produced by the enzymatic activity of CGT on the starch added to the patties were effective in decreasing the compounds responsible for the beany off-flavors (Table 1). Additionally, as the CGT added to the patties would be rendered inactive during the cooking process, it would not be considered an additive. Therefore, CDs generated by CGT in food could be an attractive strategy to mask the beany off-flavors of plant-based meat analogs and meet the increasing consumer demand for clean-label food [19].

### Sensory evaluation

We evaluated the changes in the sensory characteristics (smell and taste) of the patties with CGT treatment. First, we investigated the effect of CGT treatment on the smell of plant-based patties (Table 3). The plant-based patties were scored based on their odor (an unpleasant smell) and fragrance (a pleasant smell). Interestingly, CGT treatment significantly reduced the odor values of plant-based patties (p<0.05). In contrast, this treatment enhanced the fragrance values, albeit there were no significant differences (p>0.05). This suggested that CGT treatment could reduce the unpleasant beany odor released from plant-based patties.

**Table 3:** Smell test for plant-based patties.

	Odor	Fragrance
Control	3.83 ± 1.23^a^	2.65 ± 0.90^a^
Starch + CGT	1.22 ± 0.54^b^	4.27 ± 0.63^a^

Smell of plant-based patties was evaluated by a panel of five experts. The samples were evaluated for two attributes (odor, and fragrance) and were scored on a scale from 1 (weak) to 5 (strong). Data shows the mean ± standard deviation of the 5 expert scores. Different letters in the same row mean significant differences at

*p<0.05.

Next, the effects of CGT treatments on the taste of plant-based patties were evaluated (Table 4). The plant-based patties were evaluated based on five attributes: sweetness, sourness, saltiness, bitterness, and umami. The CGT treatment did not affect the sweetness, sourness, saltiness, bitterness, or umami of the plant-based patties (p>0.05), suggesting that the CGT treatment did not significantly affect the taste of the patties. These results demonstrate that CGT treatment could be an effective method for masking the beany off-flavor of plant-based patties without affecting the taste.

**Table 4:** Taste test for plant-based patties.

Taste	Sweetness	Sourness	Saltiness	Bitterness	Umami
Control	0.23 ± 0.05^a^	1.90 ± 0.81^a^	2.32 ± 0.96^a^	1.82 ± 0.75^a^	3.02 ± 1.12^a^
Starch + CGT	0.33 ± 0.25^a^	1.11 ± 0.58^a^	2.88 ± 1.13^a^	3.07 ± 1.29^a^	2.65 ± 0.80^a^

Taste of plant-based patties was evaluated by a panel of five experts. The samples were evaluated for five attributes (sweetness, sourness, saltiness, bitterness, and umami) and were scored on a scale from 1 (weak) to 5 (strong). Data shows the mean ± standard deviation of the 5 expert scores. Different letters in the same row mean significant differences at

*p<0.05, as determined.

In some previous studies, the addition of lower CD concentrations to soy-based foods did not affect their sensory attributes [15, 35]. It is indicated that these foods contained a quantity of volatile beany flavor compounds, well above their threshold values, compared with the addition amounts of CDs. According to Suratmen 2004, 0.5% CD-added soymilk contained hexanal, 1-octen-3-ol, and benzaldehyde concentrations that were 278-, 787-, and 8,150-fold more than the threshold value (0.05, 0.01, and 0.0004 μg/mL) [36, 37]. In the present study, CGT treatment produced about 2% CDs in plant-based patties, which was enough to reduce the beany odor by the sensory evaluation. In fact, the volatilization amounts of hexanal, 1-octen-3-ol, and benzaldehyde released from CGT-treated patties containing starch were only 0.55, 0.84, and 0.023 μg/g, that were 11-, 84-, and 57-fold more than the threshold value. Considering the individual differences in the sensitivity to the threshold and lower volatile amount than in the previous study, it is considered that the CGT treatment in this study improved the sensory characteristics of the plant-based patties.

The CGT-catalyzed reaction generally produces CDs, maltooligosaccharides, and dextrin. Maltooligosaccharides, and dextrin are known to be hydrolyzed by human salivary α-amylase, producing glucose and maltose [38, 39]. However, CGT treatment did not affect the sweetness of the plant-based patties (Table 4). It is possible that the hydrolysis by α-amylase did not provide significant sweetness as the in-mouth contact time used by the panel was extremely short (< 30 sec).

### Physical properties of meat analog patties

Generally, most meat analog products contain starch [8]. Functionally, the addition of starch improves the interaction between the protein, lipid, and water components in meat analog patties and improves the texture and consistency of the patties [8]. Moreover, starch is often used as a bulking agent in meat analog products because of its effect on textural properties [40]. Thus, we also investigated the adverse effects of partially converting starch to CDs using CGT on the physical properties of meat analog patties. Table 5 shows the TPA parameters (hardness, cohesiveness, springiness, and chewiness) of the meat analog patties. The hardness and chewiness of the patties containing starch were slightly higher than those of the control patties as the increase in the viscosity and shear of gelatinized starch by cooling enhances the hardness of the patties [40]. The hardness and chewiness of CGT-treated patties containing starch were equivalent to those of untreated patties containing starch (Table 5). These findings indicate that CGT treatment for masking beany off-flavors did not decrease the textural properties of the plant-based meat analog patties.

**Table 5:** Texture profile analysis of meat analogs. Data are presented as the mean ± standard deviation of three experiments.

	Hardness (N)	Cohesiveness	Springiness	Chewiness (N)
Non-treated patty (control)	20.6 ± 1.2^a^	0.62 ± 0.03^a^	0.65 ± 0.04^a^	8.3 ± 0.3^b^
Non-treated patty containing starch	22.5 ± 1.1^a^	0.67 ± 0.08^a^	0.70 ± 0.10^a^	10.6 ± 0.8^a^
CGT-treated patty	20.4 ± 1.3^a^	0.61 ± 0.05^a^	0.66 ± 0.06^a^	8.2 ± 0.4^b^
CGT-treated patty containing starch	21.5 ± 1.8^a^	0.65 ± 0.09^a^	0.71 ± 0.12^a^	10.0 ± 0.6^a^

Data are presented as mean values ± standard deviation of three independent experiments. Different letters in the same row mean significant differences at

*p<0.05. CGT, cyclodextrin glucanotransferase.

### Cooking loss of meat analog patties

**Fig 3:**
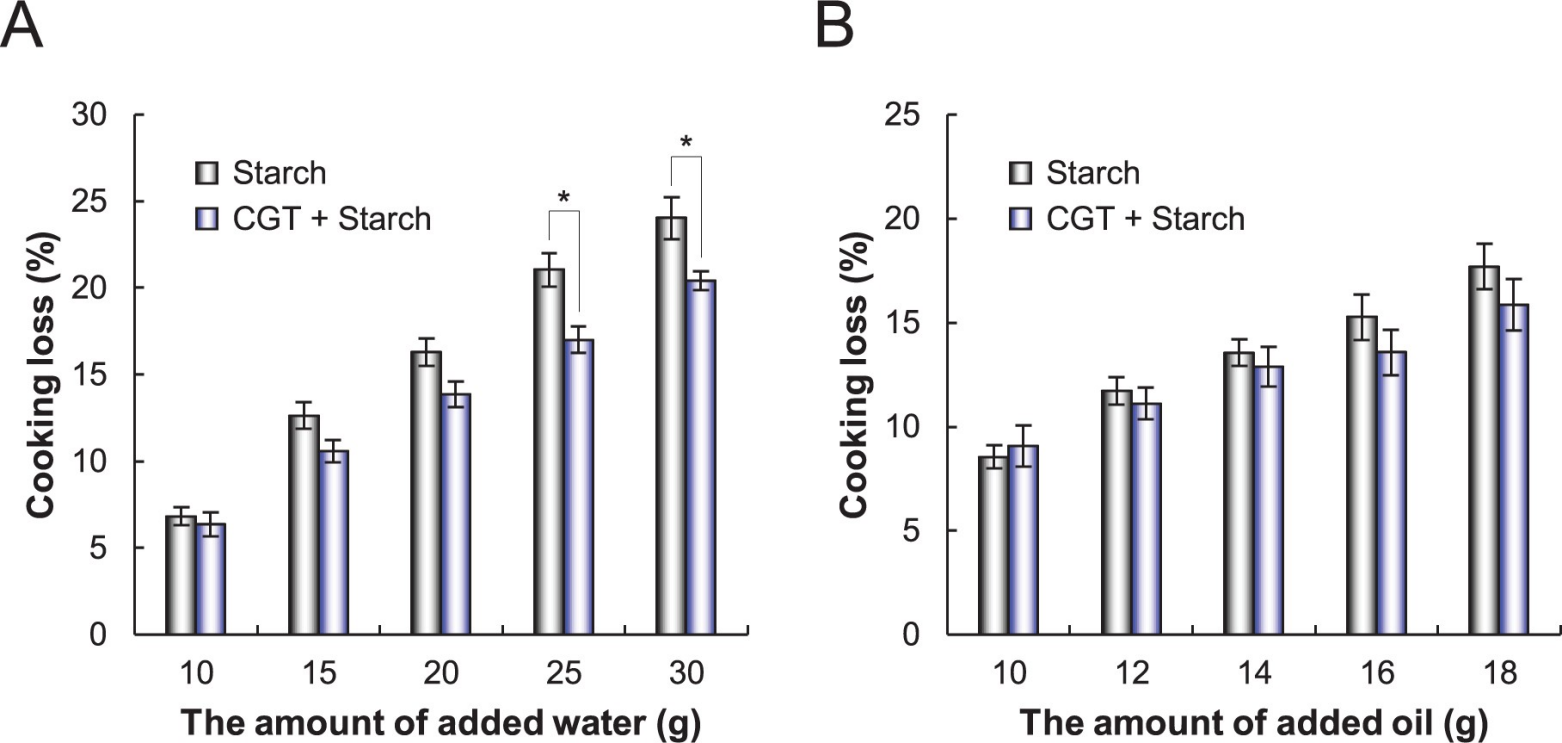
Cooking loss value of meat analog patties. (A, B) Cooking loss value was calculated as the percentage weight difference between the dough before cooking and after cooking. Meat analog patties containing different amounts of water (A) or oil (B) were treated with cyclodextrin glucanotransferase and starch and cooked. Data are presented as the mean ± standard deviation of three experiments.

Next, we investigated the adverse effects of cooking loss on meat analog patties. Cooking loss represents the degree of meat shrinkage during cooking and is an important indicator for evaluating the juiciness and yield of the final product. As the amount of water and oil increased, a typical increase in cooking loss of all patties was observed (Fig 3). Interestingly, the cooking loss of CGT-treated patties containing starch was substantially lower than that of non-treated patties containing starch under the condition of higher water content (Fig 3A). The cooking loss of CGT-treated patties containing starch was approximately 1.3-fold higher than that of non-treated patties containing starch. However, under conditions of higher oil content, the cooking loss of CGT-treated patties containing starch was also slightly lower (Fig 3B). These findings indicate that CGT treatment for masking beany off-flavors improves the cooking loss and water/oil-holding capacity of plant-based meat analog patties.

The use of MC lowers the water/oil-holding capacity [20, 41, 42]. Previous studies have reported that the cooking loss of plant-based patties containing MC is higher than that of patties containing other binders [20, 21]. This is an unacceptable phenomenon for both consumers and manufacturers. In this study, the addition of starch to MC did not affect the water-holding capacity of plant-based patties, whereas CGT treatment enhanced the water-holding capacity of patties containing starch (Fig 3A). Generally, starch gelatinization retains water, but starch retrogradation expels water as the temperature decreases, decreasing the water-holding capacity [43]. To prevent retrogradation, α-amylase treatment is used to decrease the amylose-chain length. Importantly, a previous study reported that CGT is a better antistaling enzyme than α-amylase because of its starch hydrolyzing and cyclizing activities [44]. Thus, it is possible that the partially decomposed starch and CDs formed by CGT enhanced the water-holding capacity of plant-based patties.

The CGT treatment also enhanced the oil-holding capacity of patties containing starch (Fig 3B). In a previous study, the addition of β-CD and starch to a lipid solution formed an amylose–CD–lipid complex owing to the interaction between amylose and β-CD [45]. This complex has been reported to retain lipids and retard retrogradation [43, 45]. In addition, it has been reported that the addition of α-CD helps retain fat globules in the microstructure and improves yields for animal-based products (chicken frankfurters) [46]. Thus, we investigated the synergistic effects of CD and starch on the oil-holding capacity of patties under the condition of higher oil content (data in S2 Fig). Adding both CDs and starch slightly decreased the cooking loss compared to the addition of CDs alone. These findings suggest that amylose–CD–lipid complexes formed by CGT can enhance the oil-holding capacity of plant-based patties.

## Conclusions

The generation of volatile compounds producing beany off-flavors decreases the consumer acceptability of plant-based meat products. CDs are added to plant-based meat products to mask the beany off-flavor. However, with the increasing demand for clean-label food products, decreasing the number of additives added to food products is becoming increasingly important. To mask the beany off-flavors of soy-based meat analogs while attempting to decrease the number of food additives, we used the enzymatic conversion of starch to CDs using CGT. Treating the meat analog patties containing potato starch with CGT generated α-CD and β-CD, which effectively decreased the concentration of the beany off-flavor-producing compounds, which was also confirmed by a sensory test. As CGT would be rendered inactive after cooking, it would not be considered an additive. One of the main novelties of this study is the development of a new strategy to mask the beany off-flavors of plant-based meat analogs using CGT while meeting clean-label requirements. This method would significantly help the food producers to make clean-labeled plant-based meet analogs more attractive to the demanding consumer.

## Supporting information

S1 Fig HPLC chromatography of α-, β-, and γ-cyclodextrins produced by cyclodextrin glucanotransferase. (PPTX)

S2 Fig Cooking loss value of meat analog patties containing various saccharides. Cooking loss was calculated as the percentage weight difference before and after cooking. Meat analog patties were mixed with 10 g of water, 8 g of oil, 2% methylcellulose, and 2% of each saccharide (starch, α-cyclodextrin, β-cyclodextrin, and γ-cyclodextrin). Data are presented as the mean ± standard deviation of three experiments.
(PPTX)

S1 Table Amounts of additives or enzymes added to the plant-based meat analog patties. (DOCX)
