# Supplementary figures and images for: Cyclodextrins produced by cyclodextrin glucanotransferase mask beany off-flavors in plant-based meat analogs
Source: PLoS One. 2022 Jun 3;17(6):e0269278. doi: 10.1371/journal.pone.0269278 (PMC9165781; doi:10.1371/journal.pone.0269278)

## Slide 1
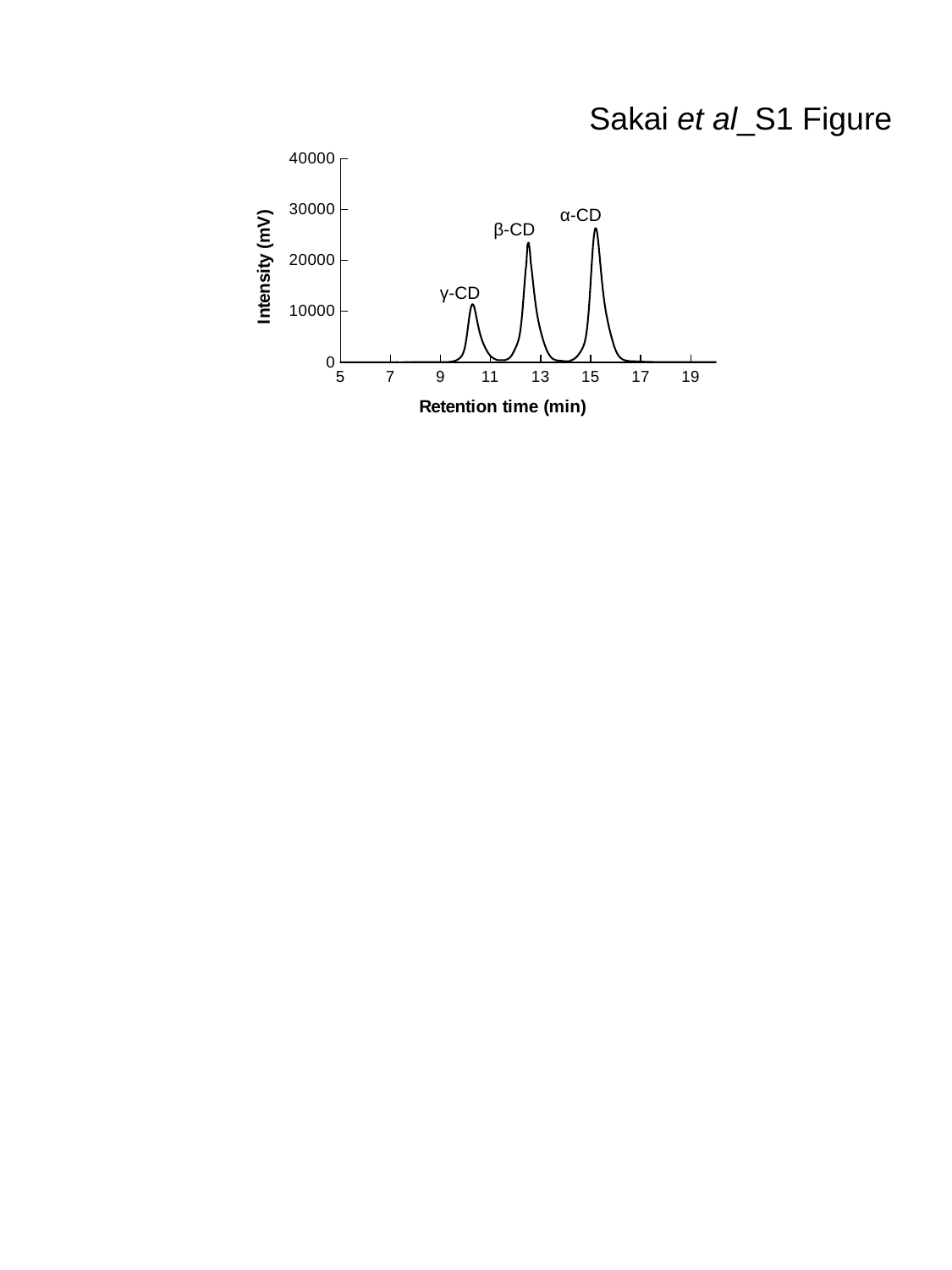

Sakai et al_S1 Figure
### Chart
| Category | |
|---|---|α-CD
β-CD
γ-CD

Supplement: S1 Fig — (PPTX) [file pone.0269278.s001.pptx]

## Slide 1
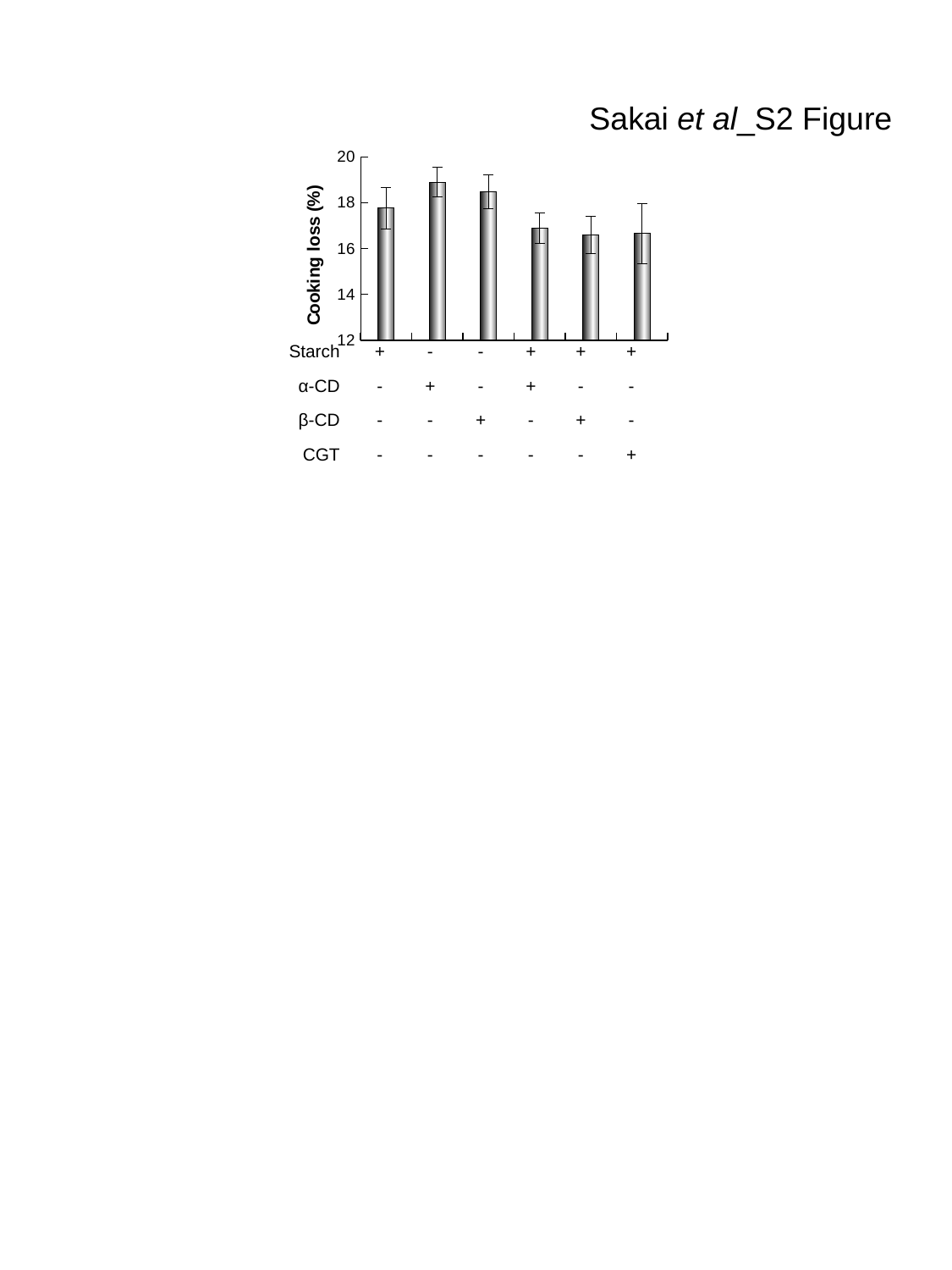

Sakai et al_S2 Figure
### Chart
| Category | 20 g oil |
|---|---|Starch
+
-
-
+
+
+
α-CD
-
+
-
+
-
-
β-CD
-
-
+
-
+
-
CGT
-
-
-
-
-
+

Supplement: S2 Fig — Cooking loss was calculated as the percentage weight difference before and after cooking. Meat analog patties were mixed with 10 g of water, 8 g of oil, 2% methylcellulose, and 2% of each saccharide (starch, α-cyclodextrin, β-cyclodextrin, and γ-cyclodextrin). Data are presented as the mean ± standard deviation of three experiments. (PPTX) [file pone.0269278.s002.pptx]
