# Supplementary material for: Cyclodextrins produced by cyclodextrin glucanotransferase mask beany off-flavors in plant-based meat analogs
Source: PLoS One. 2022 Jun 3;17(6):e0269278. doi: 10.1371/journal.pone.0269278 (PMC9165781; doi:10.1371/journal.pone.0269278)
Supplement: S1 Table — (DOCX) [file pone.0269278.s003.docx]

**S1 Table.** Amounts of additives or enzymes added to the plant-based meat analog patties.

| Sample | Wet TVP (g) | Water (g) | Oil  (g) | MC  (%) | Starch (%) | CDs  (%) | CGT  (U/g-starch) |
| --- | --- | --- | --- | --- | --- | --- | --- |
| Non-treated patty (Control) | 25 | 10 | 3 | 2 | 0 | 0 | 0 |
| Non-treated patty with starch | 25 | 10 | 3 | 2 | 2 | 0 | 0 |
| CGT-treated patty | 25 | 10 | 3 | 2 | 0 | 0 | 100 |
| CGT-treated patty with starch | 25 | 10 | 3 | 2 | 2 | 0 | 100 |
| Non-treated patty with α-CD | 25 | 10 | 3 | 2 | 0 | 5 | 0 |
| Non-treated patty with β-CD | 25 | 10 | 3 | 2 | 0 | 5 | 0 |
| Non-treated patty with γ-CD | 25 | 10 | 3 | 2 | 0 | 5 | 0 |

TVP, textured vegetable proteins; MC, methylcellulose; CDs, cyclodextrins; CGT, Cyclodextrin glucanotransferase
